# Physiological and Pathophysiological Consequences of a 25-Day Ultra-Endurance Exercise Challenge

**DOI:** 10.3389/fphys.2019.00589

**Published:** 2019-05-15

**Authors:** Nicholas B. Tiller, Scott T. Chiesa, Justin D. Roberts, Louise A. Turner, Siana Jones, Lee M. Romer

**Affiliations:** ^1^Academy of Sport and Physical Activity, Sheffield Hallam University, Sheffield, United Kingdom; ^2^Institute of Cardiovascular Science, University College London, London, United Kingdom; ^3^Cambridge Centre for Sport and Exercise Sciences, Anglia Ruskin University, Cambridge, United Kingdom; ^4^Centre for Human Performance, Exercise and Rehabilitation, Brunel University London, London, United Kingdom

**Keywords:** cardiovascular, nutrition, respiratory, ultra-endurance, ultra-marathon

## Abstract

**Background:** This case-report characterized the respiratory, cardiovascular, and nutritional/gastrointestinal (GI) responses of a trained individual to a novel ultra-endurance exercise challenge.

**Case Presentation:** A male athlete (age 45 years; $\dot{V}$O_2_max 54.0 mL⋅kg^-1^⋅min^-1^) summited 100 mountains on foot in 25 consecutive days (all elevations >600 m).

**Measures:** Laboratory measures of pulmonary function (spirometry, whole-body plethysmography, and single-breath rebreathe), respiratory muscle function (maximum static mouth-pressures), and cardiovascular structure and function (echocardiography, electrocardiography, large vessel ultrasound, and flow-mediated dilatation) were made at baseline and 48 h post-challenge. Dietary intake (four-day food diary), self-reported GI symptoms and plasma endotoxin concentrations were assessed at baseline, pre/post mid-point, pre/post end-point, and 48 h post-challenge.

**Results:** The challenge was completed in a total exercise time of 142 h (5.3 ± 2.8 h⋅d^-1^), with a distance of 1141 km (42.3 ± 43.9 km⋅d^-1^), and energy expenditure of 80460 kcal (2980 ± 1451 kcal⋅d^-1^). Relative to baseline, there were post-challenge decreases in pulmonary capacities and expiratory flows (≤34%), maximum expiratory mouth-pressure (19%), and maximum voluntary ventilation (29%). Heart rate variability deteriorated, manifesting as a 48% decrease in the root mean square of successive differences and a 70% increase in the low-frequency/high-frequency ratio. Pre- to post-challenge endotoxin concentrations were elevated by 60%, with a maximum increase of 130% after a given stage, congruent with an increased frequency and severity of GI symptoms.

**Conclusion:** The challenge resulted in pulmonary and autonomic dysfunction, endotoxaemia, and GI distress. The findings extend our understanding of the limits of physiological function and may inform medical best-practice for personnel supporting ultra-endurance events.

## Background

Ultra-endurance exercise (>6 h duration) induces substantial physiological demands on multiple body systems (Zaryski and Smith, 2005). For example, single-stage ultra-marathon can provoke a pre- to post-race decrease in magnetically evoked mouth twitch-pressure, indicative of inspiratory muscle fatigue (Wuthrich et al., 2015), and post-race reductions in pulmonary function (Vernillo et al., 2015). Studies of the cardiovascular response to Ironman triathlon and ultra-marathon have shown acute (transient) reductions in right ventricular ejection fraction (O’Keefe et al., 2012), with long-term participation causing pathological changes in cardiac structure, function, and electrical activity (for review, see George et al., 2012). Gastrointestinal (GI) distress is a commonly cited reason for ultra-marathon non-completion (de Oliveira and Burini, 2014) and is associated with the acute release of intestinal endotoxins (Jeukendrup et al., 2000), the repeated exposure to which may lead to a low-grade inflammatory state (Mach and Fuster-Botella, 2017). Collectively, the literature indicates that ultra-endurance exercise is sufficient to cause acute physiological dysfunction and chronic maladaptations of the respiratory, cardiovascular, digestive, and immune systems (Knechtle and Nikolaidis, 2018).

Participation in ultra-endurance events has steadily increased over the last 30 years (Hoffman et al., 2010). Despite the available research, no study has investigated the integrative physiological consequences of ultra-endurance exercise repeated on consecutive days. Data to this effect may inform best practice for personnel overseeing the events; i.e., medics, race directors, and volunteers. Accordingly, we implemented a series of laboratory- and field-assessments with the aim of characterizing the respiratory, cardiovascular, and nutritional/GI responses to a 25-day ultra-endurance exercise challenge contested by an experienced athlete. The unique challenge also offered an excellent opportunity to study the limits of human physiological function.

## Case Presentation

### Participant

The participant (one of four contesting the challenge) was a highly-trained male endurance athlete (age 44.8 years; mass 81.7 kg; stature 1.71 m). He had ∼20 years of experience contesting running and cycling events, had been preparing for the challenge for ∼18 months by competing in marathon and ultra-marathon running and walking events, and had a single-stage marathon season’s best of 3 h 20 min. Overarching ethical approval for the study was granted by the Sheffield Hallam University Research Ethics Committee, in collaboration with University College London, Anglia Ruskin University, and Brunel University London. Written, informed consent was provided after the participant received medical clearance from his General Practitioner.

### The Challenge

The 100-Peaks Challenge was novel in that it had not been attempted previously. The task was to summit 100 mountains (all elevations >600 m) throughout England (*n* = 45), Scotland (*n* = 45) and Wales (*n* = 10) in 25 consecutive days. All peaks were summited on foot, and the athletes cycled among the geographical regions. The team slept each night at a pre-determined base-camp which had been established at each of the challenge locations, and which comprised basic sleeping and cooking facilities. The team carried backpacks containing essential equipment and were supported by a crew of volunteers who provided food and basic medical assistance.

### Study Overview

Approximately 1 month prior to the start of the challenge, the participant completed an incremental exercise test on a motorized treadmill (h/p Cosmos Saturn, h/p Cosmos Sports & Medical GmbH, Traunstein, Germany) for the determination of peak cardiorespiratory responses. On a separate day, indices of respiratory, cardiovascular, and nutritional/GI function were assessed at resting baseline, and repeated at the first opportunity (48 h) after the challenge. Additional measures of nutritional/GI function were made pre- and post-exercise at the challenge mid- and end-points (see below).

### Respiratory Measures

Pulmonary function was assessed using a fully integrated system (Masterscreen Body, CareFusion, Hampshire, United Kingdom). First, airway resistance (s*R*aw_eff_, *R*aw_eff_) was measured using the interrupter technique. Next, pulmonary volumes and capacities (TLC, total lung capacity; RV, residual volume; FRC, functional residual capacity; and IC, inspiratory capacity) were assessed using body plethysmography. Dynamic volumes and flows (FVC, forced vital capacity; FEV_1_, forced expiratory volume in 1 s; and PEF, peak expiratory flow) were then assessed via spirometry. Alveolar diffusing capacity (DL_CO_) was estimated using the single-breath CO-rebreathe method. To quantify respiratory muscle strength, maximum static inspiratory mouth-pressure (MIP, from RV) and maximum static expiratory mouth-pressure (MEP, from TLC) were recorded using a handheld device (MicroRPM, CareFusion). Immediately after the mouth-pressure maneuvers, the participant rated his perceived intensity of respiratory muscle soreness by marking a line on a 100 mm visual analog scale (VAS) and indicated the location of soreness by shading areas on a body diagram (Mathur et al., 2010). Finally, the maximum voluntary ventilation in 12 s (MVV_12_) was assessed as an index of dynamic ventilatory capacity. All procedures followed recommended guidelines (Green et al., 2002; Macintyre et al., 2005; Miller et al., 2005; Wanger et al., 2005).

### Cardiovascular Measures

#### Cardiac

Cardiac morphology was assessed with an echocardiograph fitted with a 2–4 MHz phased array transducer (IE33, Philips, NV, United States) while the participant adopted a left-sided half-lateral decubitus position. A 12-lead electrocardiogram (ECG) running on dedicated software (CardioSoft, GE Healthcare, Milwaukee, WI, United States) was used to record cardiac rhythms and heart rate variability (HRV). The latter was displayed as root mean square of successive differences (RMSSD) and low/high frequency ratios (LF/HF). Cardiac assessments were made according to recommended standards (Parati et al., 2014; Oxborough et al., 2018).

#### Vascular

Blood pressure (systolic, diastolic, and mean arterial) was assessed using a digital automated sphygmomanometer (Omron M6, Omron Healthcare, Netherlands). Carotid intima-media thickness (cIMT) was measured in the common carotid artery using B-mode ultrasound (Zonare Z. OneUltra, Zonare Medical Systems, United States) connected to dedicated software (Carotid Analyzer, Medical Imaging Applications, Coralville, IA., United States). To calculate carotid-femoral pulse-wave velocity (PWV), arterial stiffness was measured using applanation tonometry (SphygmoCor Vx, AtCor Medical, NSW, Australia). Macrovascular and microvascular endothelial function were measured via flow-mediated dilatation (FMD; IE33, Philips, NV, United States) and endothelial peripheral arterial tonometry (EndoPAT, Itamar Medical, Israel), respectively.

### Nutritional/GI Measures

#### Dietary Intake and GI Distress

Following guidance on diary collation, meal content, portion size and fluid intake, a 4-day dietary intake was recorded at baseline, mid-point (day 13), and end-point (day 25). Nutritional analyses were performed using dedicated software (Nutritics Professional, Nutritics Ltd., Co., Dublin, Ireland). The participant wore a telemetric device (Fenix 3, Garmin, Hampshire, United Kingdom) which collected heart rate data allowing for the estimation of energy expenditure (Keytel et al., 2005). Concurrent with each dietary assessment, GI symptoms (GIS) were graded for frequency using a category scale (0 = none; 1 = mild/occasional; 3 = moderate; 5 = frequent) and severity using a 100 mm VAS. A nominal GI index (a.u, arbitrary units) was calculated for each reported symptom using the product of frequency and severity.

#### Endotoxin Concentration

At baseline, pre- and post-exercise at the mid-point (day 13), pre- and post-exercise at the end-point (day 25), and 48 h after the challenge, venous whole-blood was collected at rest into duplicate 4 mL vacutainers (K3EDTA, Greiner Bio-One GmbH, Kremsmunster, Austria). Samples were centrifuged for 10 min at 3000 rpm, with aliquoted plasma frozen at -80°C. Endotoxin concentration was quantified using an end-point chromogenic assay method (Pierce^®^ LAL Chromogenic Endotoxin Quantitation Kit, Thermo Fisher Scientific, Massachusetts, United States) using methods previously described (Roberts et al., 2016).

## Results

### Participant

Baseline assessments revealed no respiratory, cardiovascular, or blood-borne abnormalities. The maximal incremental exercise test elicited a $\dot{V}$O_2_max of 4.41 L⋅min^-1^ (54.0 mL⋅kg^-1^⋅min^-1^) and a $\dot{V}$_E_max of 152 L⋅min^-1^ (106% MVV_12_). The challenge required a total energy expenditure of 80460 kcal (2980 ± 1451 kcal⋅d^-1^). The participant completed the 100-Peaks Challenge in a total exercise time of 142 h [on foot = 106 h (75%); cycling = 36 h (25%)], and a mean exercise time of 5.3 ± 2.8 h⋅d^-1^. The total distance covered was 1141 km [on foot = 324 km (28%); cycling = 817 km (72%)], with a mean distance of 42.3 ± 43.9 km⋅d^-1^. The challenge required a total ascent of 33804 m [on foot = 27038 m (80%); cycling = 6765 m (20%)], and a mean ascent of 1252 ± 807 m⋅d^-1^.

### Respiratory Responses

**FIGURE 1:**
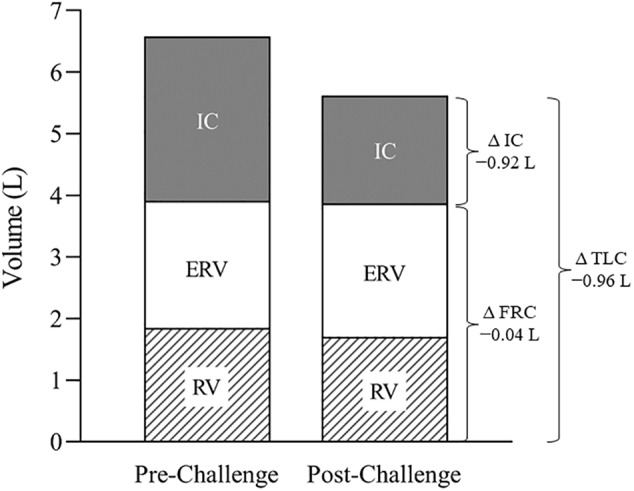
Static lung volumes and capacities at baseline and 48 h after the challenge. Note the substantial post-challenge reduction in TLC, attributable primarily to a fall in IC. TLC, total lung capacity; IC, inspiratory capacity; ERV, expiratory reserve volume; RV, residual volume; FRC, functional residual capacity.

Pulmonary and respiratory muscle function at baseline and 48 h after the challenge are summarized in Table 1. Pre- and post-challenge static lung volumes and capacities are shown in Figure 1. Baseline pulmonary function generally exceeded predicted values but remained within normal limits. At 48 h post-challenge, there were notable reductions (range 8–34%) in FVC, FEV_1_, TLC, RV, IC, PEF, MVV_12_, and metrics of airway resistance. The ratio of FEV_1_/FVC was increased slightly pre- to post-challenge (5%), with larger increases in DL_CO_ (15%). Maximum static inspiratory pressure was relatively well preserved pre- to post-challenge. By contrast, there was a substantial reduction in maximum static expiratory pressure (-19%). There were no notable changes in perceptions of respiratory muscle soreness. At day 14 of 25, the participant developed symptoms of upper-respiratory tract infection (URTI) including sore throat and mucous production that remained until the post-challenge assessment.

**Table 1:** Pulmonary and respiratory muscle function at baseline and 48 h after the challenge.

	Pre-challenge		Post-challenge	
	Absolute	% Pred	Absolute	% pred	% change
FVC (L)	4.43	102	3.88	90	–12
FEV_1_ (L)	3.72	104	3.39	96	–9
FEV_1_/FVC	0.84	106	0.88	111	+5
TLC (L)	6.58	100	5.62	86	–15
RV (L)	1.85	91	1.70	84	–8
ERV (L)	2.06	159	2.17	167	+5
FRCpleth (L)	3.91	118	3.87	117	–1
IVC (L)	4.73	106	3.92	88	–17
IC (L)	2.67	84	1.75	55	–34
PEF (L/s)	11.42	131	8.21	94	–28
MVV_12_ (L/min)	143	110	102	79	–29
s*R*aw_eff_ (kPa/s/L)	0.38	33	0.32	27	–16
*R*aw_eff_ (kPa/s)	0.09	29	0.08	25	–11
D_L,CO_ (mmol/min/kPa)	10.8	108	12.4	125	+15
MIP (cmH_2_O)	–114	100	–122	107	+7
MEP (cmH_2_O)	233	170	188	140	–19

*FVC, forced vital capacity; FEV_*1*_, forced expiratory volume in one second; TLC, total lung capacity; RV, residual volume; ERV, expiratory reserve volume; FRC, functional residual capacity; IVC, inspiratory vital capacity; IC, inspiratory capacity; PEF, peak expiratory flow; MVV_*12*_, maximum voluntary ventilation in 12 s; s*R*aw_*eff*_, specific effective airway resistance; *R*aw_*eff*_, effective airway resistance; D_*L,CO*_, carbon monoxide diffusing capacity of the lung (uncorrected for Hb); MIP, maximum inspiratory pressure; MEP, maximum expiratory pressure.*

### Cardiovascular Responses

Cardiovascular structure and function at baseline and 48 h after the challenge are summarized in Table 2. Heart rate variability assessed via RMSSD decreased by 48% (23 vs. 12 ms) and the LF/HF ratio increased by 70% (1.7 vs. 2.8). At the post-challenge assessment, the E/A wave ratio had increased by 36% (1.1 vs. 1.5). The participant exhibited no noteworthy changes in any other index of cardiovascular structure or function.

**Table 2:** Cardiovascular structure and function at baseline and 48 h after the challenge.

	Pre-challenge	Post-challenge	% change
**Cardiac**			
Fractional shortening (%)	29	26	–10
LA diameter (cm)	4.5	4.3	–4
E/A wave ratio	1.1	1.5	+36
TAPSE (cm)	3.4	3.3	–3
**Autonomic**			
HR (b⋅min^-1^)	52	50	–4
HRV			
RMSSD (ms)	23	12	–48
LF/HF	1.66	2.82	+70
**Vascular**			
cIMT (mm)	0.48	0.47	–2
PWV (m/s)	7.0	7.7	+10
FMD (%)	3.5	3.2	–9
RHI	0.44	0.52	+18
**Blood Pressure**			
SBP (mmHg)	123	121	–2
DBP (mmHg)	73	68	–7
MAP (mmHg)	90	85	–6

*LA, left atrium; E/A wave ratio, the ratio of early diastolic peak flow velocity (E) to late diastolic peak flow velocity (A); TAPSE, tricuspid annular plane systolic excursion; HR, heart rate; HRV, heart rate variability; RMSSD, root mean square of successive differences; LF/HF, low frequency/high frequency; cIMT, carotid intima-media thickness; PWV, pulse wave velocity; FMD, flow mediated dilatation; RHI, reactive hyperaemia index; SBP, systolic blood pressure; DBP, diastolic blood pressure; MAP, mean arterial pressure.*

### Nutritional Intake and GI Responses

Nutritional intake and GI responses are summarized in Table 3. As expected, energy intake increased substantially throughout the challenge via progressive increases in carbohydrate ingestion (partly from refined sugars) and dietary fat. Despite the increase in energy intake, body mass was maintained pre- to post-challenge (81.7 vs. 81.4 kg). At the challenge end-point (day 25) GIS had increased from 35 to 127 a.u., reflecting both increased symptom frequency and severity. Endotoxin concentration was considered normal at resting baseline (10.9 pg⋅ml^-1^; expected range 3–10 pg⋅ml^-1^), increased modestly by the mid-point (17.1 pg⋅ml^-1^), and remained above baseline at 48 h post-challenge (17.6 pg⋅ml^-1^). There were transient increases in endotoxin concentration after a given bout of exercise when assessed at the challenge mid- and end-points (25.5 and 24.3 pg⋅ml^-1^, respectively).

**Table 3:** Nutritional intake (4-day average) and GI responses at baseline, mid-point (day 13), and end-point (day 25).

	Pre-challenge	Mid-point	End-point	% change^∗^
Body mass (kg)	81.7	81.7	81.4	<1
Energy (kcal/d)	2008	3814	4558	+127
Energy (kcal/kg/d)	24.6	46.7	56.0	+127
Carbohydrate (g/d)	156	446	581	+272
Carbohydrate (g/kg/d)	1.9	5.5	7.1	+274
Sugar (g/d)	66	160	198	+200
Sugar (g/kg/d)	0.8	2.0	2.4	+200
Protein (g/d)	157	179	185	+18
Protein (g/kg/d)	1.9	2.2	2.3	+21
Fat (g/d)	84	146	166	+98
Fat (g/kg/d)	1.0	1.8	2.0	+100
Water (mL)	1821	6520	4271	+135
Sodium (mg)	3184	5614	5622	+77
Potassium (mg)	4564	4926	5488	+20
Magnesium (mg)	672	667	721	+7
Zinc (mg)	13.9	36.9	32.9	+137
Vitamin C (mg)	161	1009	778	+383
Number of GIS reported	2	3	9	+350
GIS frequency	6	10	27	+350
GIS severity (mm)	100	85	370	+270
Mean GIS Index (a.u.)	35	20	127	+263

*GIS, gastrointestinal symptoms. GIS index based on mean data for frequency × severity for each reported symptom. ^∗^% change represents end-point (day 25) relative to pre-challenge.*

## Discussion

This is the first report of the integrative physiological response of a trained athlete to a novel ultra-endurance stage-race. At 48 h post-challenge, the principal observations were: (i) reductions in several metrics of pulmonary function and a substantial pre- to post-challenge decrease in maximum expiratory pressure; (ii) a substantial reduction in HRV; and (iii) large increases in calorie intake (congruent with metabolic demands), GIS prevalence, and endotoxin concentration. Collectively, these observations suggest that 25 days of endurance exercise has the potential to induce long-lasting respiratory, cardiovascular, and GI dysfunction.

### Respiratory Responses

The post-challenge respiratory assessments revealed a restrictive pattern characterized by a fall in attainable lung volumes and capacities (see Table 1). Although post-challenge FVC and FEV_1_ were both reduced (9–12%), the FEV_1_/FVC ratio was elevated (5%), and exceeded normal values, suggesting an elevated expiratory flow relative to lung volume. That RV and ERV were relatively well preserved while IC decreased (34%) indicates that the decrease in FVC was primarily the result of an inability to fully expand the lung on inspiration (Figure 1). Moreover, the variability of IC was similar pre- vs. post-challenge (data not shown), thereby discounting a diminished inspiratory effort. Moreover, there was no evidence of inspiratory muscle fatigue (MIP -114 vs. -122 cmH_2_O). We propose, therefore, that the restricted lung expansion was attributable to reduced lung compliance and/or increased ribcage stiffness, the causes of which require further study.

We also observed a substantial (19%) pre- to post-challenge reduction in maximum expiratory pressure (MEP), the magnitude of which was similar to that noted following marathon (15%, Ross et al., 2008), and single-stage ultra-marathon (21%, Wuthrich et al., 2015). Importantly, our study is the first to assess respiratory muscle function in response to ultra-endurance stage-racing. The pre- to post-challenge reduction in MEP is suggestive of exercise-induced expiratory muscle fatigue. Given that such fatigue typically recovers within a few hours of exercise (Romer and Polkey, 2008), the long-lasting (48 h) reduction in MEP might have been caused by mechanical changes in the expiratory muscles, including sarcolemmal disruption, myofibrillar disorganization, and z-band streaming (Reid et al., 2001). However, there are several lines of evidence that the observed decrease in MEP might have been largely independent of fatigue. First, the decrease in expiratory mouth-pressure occurred in the absence of any change in the perception of respiratory muscle soreness, thereby bringing into question the notion of respiratory muscle damage. Moreover, the aforementioned decrease in IC (and hence TLC) would be expected to attenuate MEP due to a decrease in passive recoil of the respiratory system and, in particular, a decrease in the maximal pressure-generating capacity of the expiratory muscles resulting from alterations in length-tension characteristics. Indeed, using published data (Rahn et al., 1946), the observed reduction in vital capacity (12%) was predicted to evoke a decrease in MEP of 14%. Accordingly, our data indicate that a large proportion (up to 70%) of the reduction in MEP was independent of expiratory muscle fatigue.

Finally, we noted large pre- to post-challenge reductions in expiratory airflow (i.e., FEV_1_, -9%; PEF, -28%). While such an obstructive pattern might have been associated with the URTI that remained throughout the post-challenge assessment, this appears unlikely given that airway resistance (*R*aw_eff_ and s*R*aw_eff_) had decreased pre- to post-challenge. Airflow during spirometry is dependent on both airway resistance and the driving pressure of thoracic muscles (Hayes and Kraman, 2009); as such, a more likely explanation for the reductions in FEV_1_ and PEF is a diminished thoracic driving pressure owing to a failure to start the maneuver from “true” TLC. Despite marked reductions in pulmonary and respiratory muscle function, the values tended to remain within normal limits and, therefore were unlikely to pose an acute clinical concern.

### Cardiovascular Responses

The principal finding from the cardiovascular assessment was a post-challenge decrease in RMSSD (48%) and an increase in the LF/HF ratio (70%), relative to baseline. These findings are in agreement with other reports of autonomic dysfunction following both endurance and ultra-endurance exercise (Gratze et al., 2005; Scott et al., 2009). When considered alongside our observation of a post-challenge decrease in airway resistance (see section “Respiratory Responses*”*), our findings support the notion that there might have been a withdrawal of parasympathetic control and a subsequent increase in sympathetic cardiac modulation that persisted for at least 48 h post-challenge (Gratze et al., 2005; Scott et al., 2009). Although autonomic dysfunction may lead to an abnormal cardiovascular response and reduced exercise capacity (for review, see Fu and Levine, 2013), the phenomenon requires further study in relation to ultra-endurance exercise.

Baseline right ventricular contractility (TAPSE) far exceeded the reference value (3.4 vs. 1.6 cm; Koestenberger et al., 2009), and was likely the result of chronic training-induced adaptations. Blood pressure and measures of arterial structure and stiffness (cIMT and PWV) were as expected for the participant’s age. Flow-mediated dilatation and reactive hyperaemia index (measures of macro- and microvascular endothelial function, respectively) were at the lower end of age-predicted values (Yan et al., 2005), although it is unclear whether these parameters were associated with endothelial dysfunction, *per se*, or were the result of conduit artery structural-remodeling in response to chronic training (Green et al., 2012). A brachial diameter of 0.44 mm, similar to that previously reported in trained athletes of a similar age to the present case (Montero et al., 2014), suggests that the slightly lower-than-expected flow-mediated dilatation might have been a result of training-induced remodeling of the brachial artery, but other factors (e.g., oxidative stress resulting from high training loads) cannot be discounted. We noted a post-challenge increase in the E/A ratio, indicating an improvement in diastolic function; however, values remained within normal limits.

We observed no other meaningful pre- to post-challenge changes in cardiovascular structure or function; i.e., post-challenge values tended to remain within normal limits. This is contrary to earlier reports of transient right ventricular dysfunction and reduced large artery compliance after endurance exercise (La Gerche et al., 2012) and ultra-endurance exercise (Bonsignore et al., 2017), respectively. In general, the existing literature pertaining to acute and/or chronic changes in vascular structure and function in endurance athletes report equivocal findings (Vlachopoulos et al., 2010; Green et al., 2012; Burr et al., 2014; Radtke et al., 2014). The discrepancies among studies are likely due to methodological differences such as the timing of post-exercise measurements and the broad range of exercise intensities and durations. Indeed, while the current challenge was extreme in scope (i.e., cumulative exercise time, distance, and ascent), the daily exercise duration and intensity may not have induced the cardiovascular strain necessary to influence vessel structure and function.

### Nutritional and GI Responses

As expected, energy consumption increased progressively during the challenge when expressed relative to habitual intake. In line with the present findings, previous studies report that completion of ultra-endurance events is associated with caloric and carbohydrate-rich eating regimens as well as high sodium and fluid intakes (Glace B.W. et al., 2002; Paulin et al., 2015). Nevertheless, sudden caloric “overload” (e.g., ingestion of hyperosmolar solutions) and changes in food quality (notably fat) during multi-day events can alter microbiota diversity and GI absorptive characteristics, leading to increased GIS (e.g., discomfort, sense of fullness, and urgency to defecate) (Glace B. et al., 2002; de Oliveira and Burini, 2014). In the present case, energy intake was congruent with worsening GIS from the mid-point onward, but this did not appear to critically impede performance. Broader research indicates that prior exercise training may offer a degree of “tolerance” to such symptoms, likely due to both behavioral mechanisms (i.e., greater experience and practice with individualized nutrition regimens) and physiological mechanisms (i.e., stronger immunity to endotoxins in more highly trained runners) (Brock-Utne et al., 1988).

In cohort studies, exercise-induced GI hypoperfusion has been shown to increase phosphorylation enzyme activity, provoking transient disruption of tight junction proteins (van Wijck et al., 2011; Zuhl et al., 2014). Enhanced luminal paracellular transport and/or ischaemia-associated mucosal damage may increase the prevalence of systemic lipopolysaccharides (LPS) and symptoms associated with endotoxaemia. Although acute high-intensity exercise is associated with increased luminal permeability (Pals et al., 1997), less is known about prolonged, repetitive, and low-intensity ultra-endurance exercise. In this case-study, the participant exhibited mild-to-moderate endotoxaemia (endotoxin concentrations reaching 25.5 pg⋅ml^-1^). Previous studies in trained endurance athletes have reported mild (5–15 pg⋅ml^-1^; Jeukendrup et al., 2000) to high (>100 pg⋅ml^-1^; Brock-Utne et al., 1988) endotoxin concentrations. Collectively, these findings are indicative of variable, event-specific and/or individual endotoxaemic responses.

Elevated endotoxin concentrations have been associated with low-grade cytokinemia (e.g., IL-6, C-reactive protein), possibly leading to immunosuppression, and increased prevalence of URTI (for review, see Mach and Fuster-Botella, 2017). It is possible, therefore, that endotoxaemia in our participant was related to the URTI that manifested at day 14 and remained until the post-challenge assessments. Indeed, athletes are more prone to URTI because of the physiological stress of exercise combined with an imbalanced diet, sleep disturbance, and exposure to environmental extremes (Thomas and Ockhuizen, 2012), all of which manifested in the present challenge. While recognizing the methodological limitations of endotoxin unit quantification, the present data indicate that GI responses to ultra-endurance exercise are transient and demonstrate the remarkable adaptive nature of the GI-tract to prolonged, multi-day exercise (Gisolfi, 2000).

In conclusion, this case-report provides novel data on the integrative physiological response of a trained athlete to a 25-day ultra-endurance exercise challenge. Relative to baseline, the participant exhibited marked post-challenge reductions in pulmonary function, and a decrease in maximum expiratory pressure that was only moderately attributable to expiratory muscle fatigue. Although the post-challenge pulmonary function remained above the lower-limits of normal, the same response in an individual with a pre-existing respiratory disorder (e.g., asthma) may be clinically meaningful. There was evidence of autonomic dysfunction which might influence the cardiovascular response and impact on exercise capacity. Finally, there was mild-to-moderate endotoxaemia, progressive GI distress, and symptoms of URTI likely related to immunosuppression. Our data underscore the importance of robust baseline physiological function prior to participation in such a challenge. More research is needed to elucidate the implications of chronic participation in ultra-endurance exercise for physiological function.

## Ethics Statement

The study was approved by the Sheffield Hallam University Faculty Research Ethics Committee, and the subject provided written, informed consent.

## Author Contributions

NT, SC, and JR conceived and designed the study. NT, SC, JR, LT, and SJ performed the data collection and analysis. NT, SC, JR, and LR interpreted the results, drafted, edited, and revised the manuscript. All authors approved the final draft of the manuscript.

## Conflict of Interest Statement

The authors declare that the research was conducted in the absence of any commercial or financial relationships that could be construed as a potential conflict of interest.
